# Glucagon-like peptide receptor agonists attenuate advanced glycation end products-induced inflammation in rat mesangial cells

**DOI:** 10.1186/s40360-017-0172-3

**Published:** 2017-10-24

**Authors:** Jui-Ting Chang, Yao-Jen Liang, Chia-Yu Hsu, Chao-Yi Chen, Po-Jung Chen, Yi-Feng Yang, Yen-Lin Chen, Dee Pei, Jin-Biou Chang, Jyh-Gang Leu

**Affiliations:** 1 Division of Nephrology, Department of Internal Medicine, Shin Kong Wu Ho-Su Memorial Hospital, Taipei, Taiwan; 2 Department and Institute of Life Science, Fu-Jen Catholic University, New Taipei, Taiwan; 3 Graduate Institute of Applied Science and Engineering, Fu-Jen Catholic University, New Taipei, Taiwan; 4 Department of Pathology, Cardinal Tien Hospital, Medical School, Fu Jen Catholic University, New Taipei City, Taiwan; 5 Division of Endocrinology and Metabolism, Department of Internal Medicine, Cardinal Tien Hospital, Medical School, Fu Jen Catholic University, New Taipei City, Taiwan; 6 Fu-Jen Catholic University School of Medicine, No. 510, Zhongzheng Road, Xinzhuang District, New Taipei City, 24205 Taiwan; 7 Department of Pathology, National Defense Medical Center, Division of Clinical Pathology, Tri-Service General Hospital, Taipei, Taiwan

**Keywords:** Diabetic nephropathy, Glucagon-like peptide, Mesangial cell, PPAR delta, Rage

## Abstract

**Background:**

Hyperglycemia-induced advanced glycation end products (AGEs) and receptor for AGEs (RAGE) production play major roles in progression of diabetic nephropathy. Anti-RAGE effect of peroxisome proliferator-activated receptor-delta (PPARδ) agonists was shown in previous studies. PPARδ agonists also stimulate glucagon-like peptide-1 (GLP-1) secretion from human intestinal cells.

**Methods:**

In this study, the individual and synergic anti-inflammatory effects of GLP-1 receptor (exendin-4) and PPARδ (L-165,041) agonists in AGE-treated rat mesangial cells (RMC) were investigated.

**Results:**

The results showed both exendin-4 and L-165,041 significantly attenuated AGE-induced IL-6 and TNF-α production, RAGE expression, and cell death in RMC. Similar anti-inflammatory potency was seen between 0.3 nM exendin-4 and 1 μM L-165,041. Synergic effect of exendin-4 and L-165,041 was shown in inhibiting cytokines production, but not in inhibiting RAGE expression or cell death.

**Conclusions:**

These results suggest that both GLP-1 receptor and PPARδ agonists have anti-inflammatory effect on AGE-treated rat mesangial cells.

## Background

Diabetic nephropathy is the leading cause of end-stage renal disease in most developed countries [1]. Large clinical trials revealed that strict control of blood glucose significantly reduced the development and progression of diabetic nephropathy in both type 1 and type 2 diabetes [2]. High serum glucose levels in diabetic patients stimulate the production of advanced glycation end products (AGEs), the transmembranous receptor for AGEs (RAGE), and various RAGE ligands in kidney cells. Ligand-induced activation of RAGE triggers numerous different signaling pathways, enhances oxidative stress, increases production of inflammatory substances, and promotes fibrosis formation [3–5]. Mesangial cells increased fibronectin production and decreased proliferation in growing on AGE-modified matrix protein. Mesangial matrix expansion is one characteristic of diabetic nephropathy [6]. The anti-RAGE and anti-inflammatory therapies in mesangial cell may prevent the progression of diabetic nephropathy.

The peroxisome proliferator-activated receptor superfamily (PPARs) comprises a class of nuclear receptors with significant effects in regulating multiple cellular pathways. Activation of PPARs is through a ligand-dependent transactivation mechanism, modulating the transcriptional activity of various target genes. PPAR family comprises of three subtypes: PPARα, PPARγ, and PPARδ. PPAR agonists are widely used in clinical therapy to enhance serum glucose control. The renoprotective effect of PPARδ agonist on diabetic nephropathy was studied recently. A PPARδ agonist, L-165,041, significantly decreased serum tumor necrosis factor-α (TNF-α), interleukin-6 (IL-6), and interleukin-1 (IL-1) levels, and kidney RAGE expression in streptozotocin-treated (STZ) diabetic mice [5]. Another PPARδ agonist, GW0742, decreased urinary albumin excretion, macrophage infiltration, mesangial matrix accumulation, and expression of inflammatory mediators in kidney tissue of STZ mice [7]. PPARδ agonists also attenuated AGE-induced pro-inflammatory cytokine production, RAGE expression, nuclear factor-*κ*B pathway activation, oxidative stress, and cells apoptosis in human embryonic kidney 293 (HEK) cells [7].

Glucagon-like peptide-1 (GLP-1) is a gut incretin hormone secreted in response to food intake and augments glucose-induced insulin release from pancreatic cells [8]. GLP-1 was shown to attenuate AGE-induced RAGE mRNA expression and oxidative stress in human proximal tubular epithelial [9] and mesangial cells [10]. GLP-1 receptor agonists are being widely used in clinical treatment for type 2 diabetes. The GLP-1 receptor agonist, exendin-4, was shown to delay the progression of diabetic nephropathy in male *db/db* mice [9]. Urinary albumin excretion, mesangial matrix expansion, transforming growth factor-β1 (TGF-β1) expression, and type IV collagen accumulation in mouse kidney were all significantly decreased by exendin-4. All these effects were independent to serum fasting blood glucose and glycated hemoglobin levels. Exendin-4 also inhibits high glucose (30 mmol/L)-induced TGF-β1 and connective tissue growth factor (CTGF) expression in human mesangial cells [11].

Recently, PPARδ agonists were found having ability to enhance glucose- and bile acid–induced GLP-1 release by intestinal L cells in vitro and ex vivo in human jejunum. This phenomenon was not seen in PPARδ-deficient mice [12]. The aim of this study is to identify the renoprotective mechanisms of GLP-1 receptor agonists, and compare the potency between GLP-1 receptor and PPARδ agonists in kidney cells.

## Methods

### Preparation and characterization of AGEs

AGEs were produced according to the method of Vinson and Howard with slight modifications, by incubating 10 mg/mL of fatty acid-free bovine serum albumin (BSA) with 25 mM glyceraldehydes and 1 mM diethylenetriaminepentaacetic acid in 0.1 M phosphate-buffered saline (pH 7.4) at 37 °C for 7 days. Unbound sugars were removed by dialysis in 10 mM phosphate-buffered saline (pH 7.4) for 24 h. The protein content was determined by Lowry assay, using BSA as the standard. Estimation of AGE content by spectrofluorometry with excitation wavelength of 390 nm and emission wavelength of 450 nm revealed a 6.5-fold increase in fluorescence for AGE–BSA compared to control BSA. According to the concentration pre-test experiments, a concentration of 200 uM of AGE was chosen for following experiments [7].

### Cell culture and reagents

Rat mesangial cells (RMC) were incubated in low-glucose (5.56 mM) media DMEM (Dulbecoo’s modified eagle’s medium; GIBCO™ 10567) with 10% fetal bovine serum at 37 °C in humidified atmosphere with 5% CO2. When the mesangial cells showed aggregation growth, gradually fused and passaged them, switched to serum-free medium. The experiments were performed after 4–6 passages and 80% confluence. L-165,041 and exendin are from Sigma.

### Measurement of tumor necrosis factor-α (TNF-α) and interleukin-6 (IL-6)

The cells were treated with AGE for 18 h with or without L-165,041 or exendin. The TNF-α and IL-6 levels in cell supernatants were measured by enzyme-linked immunosorbent assay (ELISA) kits (Peprotech Inc., Rocky Hill, NJ). Supernatants were diluted in the ratio of 1:30 before assay. The reaction products were measured at 450 nm with a microplate reader.

### RNA isolation and reverse transcription

Total cellular RNA was isolated from RMC using the single step acid guanidinium thiocyanate/phenol/chloroform extraction method. For reverse transcription, 1 μg of RNA was incubated with 200 U of HiScript I reverse transcriptase (Bionovas Biotechnology, Toronto, Canada) in a buffer containing a final concentration of 20 mmol/l Tris/HCl (pH 7.8), 100 mmol/l NaCl, 0.1 mmol/l EDTA, 1 mmol/l DTT, 50% glycerol, 2.5 mol/l poly (dT)_12–18_ oligomer, and 0.5 mmol/l of each dNTP at a final volume of 20 μL. The reaction mixture was incubated at 45 °C for 1 h and then at 70 °C for 15 min to inactivate the enzyme. The produced cDNA was used to generate DNA product by polymerase chain reaction (PCR).

### Real-time PCR

The cDNA had a 10-fold dilution in nuclease-free water and was used for the Smart Quant Green Master Mix (Protech Technology Enterprise Co., Taipei, Taiwan): 2 μL of cDNA solution, 0.5 μmol/L primers, 5 mmol/L magnesium chloride, and 2 μL of Master SYBRGreen in nuclease-free water with a final volume of 20 μL. The primers used for PCR were: RAGE: forward, 5′AAGCCCCTGGTGCCTAATGAG3′, reverse, 5′CACCAATTGGACCTCCTCCA3′; GLP-1: forward, 5′CATTCACAGGGCACATTCACC3′, reverse, 5’ACCAGCCAAGCAATGAATTCCTT3’; GAPDH: forward, 5’AGACAGCCGCATCTTCTTGT3’, reverse, 5’TTCCCATTCTCAGCCTTGAC3’. The initial denaturizing phase was 5 min at 95 °C followed by an amplification phase as detailed below: denaturation at 95 °C for 10 s; annealing at 55 °C for 10 s; elongation at 72 °C for 15 s and detection at 79 °C for 45 cycles. Amplification, fluorescence detection, and post-processing calculation were performed using the ABI step1 apparatus. Individual PCR product was analyzed for DNA sequence to confirm the purity of the product.

### RNA interference

Rat mesangial cells were transfected with 800 ng PPARδ annealed siRNA oligonucleotide (sc-36,306) or siRNA of green fluorescent protein (GFP). PPARδ siRNA is a pool of 3 target-specific 20–25 nt siRNA according to a computer program provided by Santa Cruz. The negative control, GFP siRNA was used: sense: 5′–GGCUACGUCCAGGAGCGCACC; antisense: 5′–UGCGCUCCUGGACGUAGCCUU (Dharmacon Inc., Lafayette, CO, USA). After overnight incubation, cells were treated with AGE, L-165,041, or exendin-4, and subjected to analysis by Western blotting.

### Western blot analysis

Total protein samples were mixed with sample buffer, boiled for 5 min, separated by 10% SDS-PAGE under denaturing conditions, and electroblotted to nitrocellulose membranes (Amersham Pharmacia Biotech, CB, UK). The nitrocellulose membranes were blocked in blocking buffer, incubated with human anti-RAGE, anti-Nox4, anti-PKA, and anti-HO-1 (Santa Cruz Biotechnology Inc., CA) antibodies, washed, and incubated with horseradish peroxidase-conjugated secondary antibodies. Signals were visualized by enhance chemiluminescent detection.

### Cell viability test

RMC were seeded onto 96-well plates in medium containing 10% FBS and then incubated with L-165,041 or exendin, or control medium alone in 5% CO_2_ for 18 h at 37 °C. The cell viability was determined using the WST-8 assay kit (Kishida Chemical Co., Ltd. JAPAN) following the manufacturer’s instructions.

### Statistical analysis

The data were expressed as mean ± SEM. A Tukey test was used for comparing parametric variables between the two groups, while ANOVA with repeat measurement design was used for time-course changes. Statistical significance was evaluated by Tukey test (GraphPad Software Inc., San Diego, CA). A *p*-value of less than 0.05 was considered statistically significant.

## Results

### PPARδ and GLP-1 receptor agonists inhibit AGE-induced IL-6 and TNF-α production

**Fig. 1:**
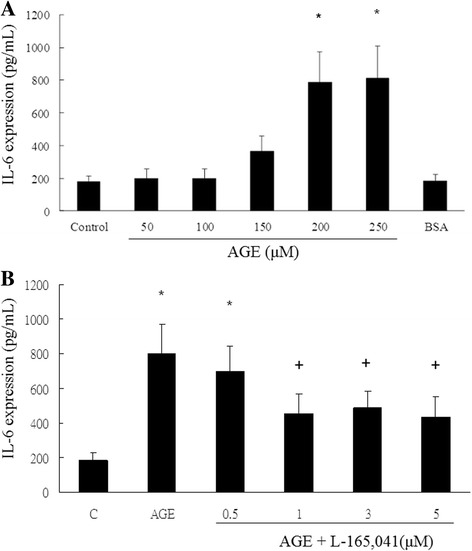
L-165,041 attenuates AGE-induced interleukin-6 (IL-6) production in rat mesangial cells. ELISA was used to measure IL-6 concentrations in cell culture supernatants. **a** AGE stimulated IL-6 production with a dose-dependent manner. A concentration of 200 μM of AGE was chosen for following experiments. **b** The AGE-induced IL-6 production was inhibited L-165,041 with a dose-dependent manner. A concentration of 1 μM was chosen for following experiments. (*N* = 6) *P < 0.05 when compared to control. +P < 0.05 when compared to AGE group

**Fig. 2:**
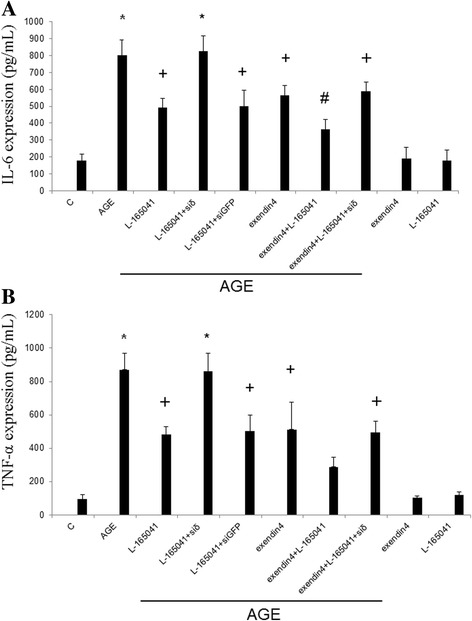
Exendin-4 and L-165,041 inhibit AGE-induced IL-6 (**a**) and TNF-α (**b**) production in RMC. The inhibitory action of L-165,041 was reversed by siRNA of PPARδ. L-165,041 and exendin-4 showed synergic effect in inhibiting IL-6 and TNF-α production. L-165,041 or exendin-4 alone had no influence on IL-6 or TNF-α production in the absence of AGE. (N = 6) *P < 0.05 when compared to control. +P < 0.05 when compared to AGE group. #P < 0.05 when compared to L-165,041 or exendin-4 group

Rat mesangial cells (RMC) were treated by serial dilutions of AGE. AGE stimulated IL-6 production in RMC with a dose-dependent manner (Fig.1a). A concentration of 200 μM of AGE was chosen for following experiments. The AGE-induced IL-6 production was inhibited by the PPARδ agonist, L-165,041, with a dose-dependent manner (Fig.1b). A concentration of 1 μM of L-165,041 was chosen for following experiments [7]. Pretreatment with L-165,041 or a GLP-1 receptor agonist, exendin-4, significantly attenuated AGE-induced IL-6 (Fig. 2a) and TNF-α (Fig. 2b) production in RMC. The inhibitory action of L-165,041 was reversed by siRNA of PPARδ. L-165,041 and exendin-4 showed synergic effect in inhibiting IL-6 and TNF-α production. L-165,041 or exendin-4 alone, however, had no influence on IL-6 or TNF-α production in the absence of AGE.

### PPARδ and GLP-1 receptor agonists inhibit AGE-induced RAGE expression

**Fig. 3:**
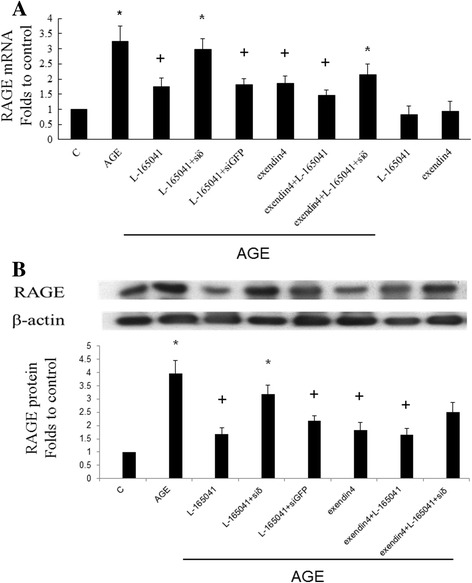
Exendin-4 and L-165,041 inhibit AGE-induced RAGE mRNA (**a**) and protein (**b**) expression in RMC. AGE significantly induced RAGE mRNA and protein expression. Both exendin-4 and L-165,041 significantly attenuated AGE-induced RAGE expression. The inhibitory action of L-165,041 was reversed by siRNA of PPARδ. No synergic effect between L-165,041 and exendin-4 in inhibiting RAGE expression was noted. L-165,041 or exendin-4 alone did not change RAGE expression in the absence of AGE. (N = 6) *P < 0.05 when compared to control. +P < 0.05 when compared to AGE group

RAGE is a key factor for causing diabetic nephropathy. We examined the effect of PPARδ and GLP-1 receptor agonists against AGE-induced RAGE upregulation. As shown in Fig. 3, AGE significantly induced RAGE mRNA (Fig. 3a) and protein expressions (Fig. 3b) in RMC. Both L-165,041 and exendin significantly attenuated AGE-induced RAGE expression. The inhibitory action of L-165,041 was reversed by siRNA of PPARδ. No synergic effect between L-165,041 and exendin in inhibiting RAGE expression was noted. L-165,041 or exendin alone did not change RAGE expression in the absence of AGE.

### The anti- oxidation effects of PPARδ and GLP-1 receptor agonists

**Fig. 4:**
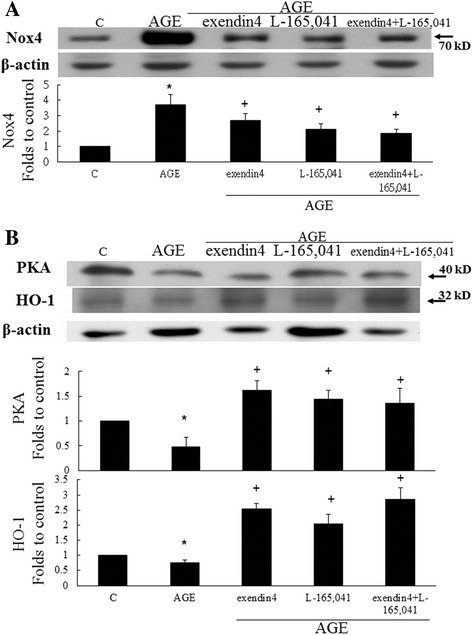
The effects of L-165,041 and exendin treatment on the NAPDH oxidase, PKA and HO-1 expressions. **a** AGE significantly increased the Nox4 expressions. Both L-165,041 and exendin significantly decreased AGE-induced Nox4. **b** After L-165,041 and exendin treatment, the levels of AGE-inhibited PKA and HO-1 expression significantly increased in mesangial cells. (N = 6) *P < 0.05 when compared to control. +P < 0.05 when compared to AGE group

The expressions of NADPH oxidase is a key important source of ROS. After AGE treatment, Nox4 expressions were significantly increased. However, L-165,041 and/or exendin significantly decreased AGE-induced Nox4 (Fig. 4a). Furthermore, both L-165,041 and exendin treatment reversed the AGE-decreased PKA expression in mesangial cells (Fig. 4b). The level of inhibitory did not show significantly synergic effects after L-165,041 and exendin treated together. It is known that, heme oxygenase-1 (HO-1) is an anti-oxidant and anti-apoptotic substance. Exendin treatment increased the AGE-decreased HO-1 expression better than L-165,041 treatment in mesangial cells (Fig. 4b).

### PPARδ and GLP-1 receptor agonists attenuate AGE-induced cell death

**Fig. 5:**
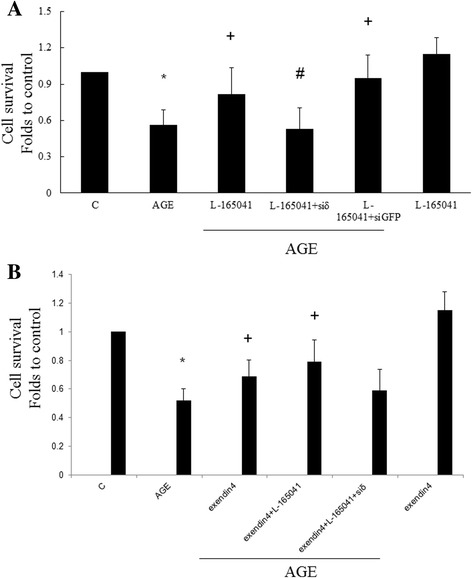
Exendin-4 and L-165,041 inhibit AGE-induced cell death of RMC. AGE enhanced death of RMC. Both exendin-4 (**a**) and L-165,041 (**b**) significantly attenuated AGE-induced cell death. The effect of L-165,041 was reversed by siRNA of PPARδ. No synergic effect between exendin-4 and L-165,041 in inhibiting AGE-induced cell death was noted. Exendin-4 or L-165,041 alone showed no influence on cell survival in the absence of AGE. (N = 6) *P < 0.05 when compared to control. +P < 0.05 when compared to AGE group

Previous studies showed that L-165,041 attenuated high glucose-induced apoptosis in human embryonic kidney (HEK) and mesangial cells (HMC). In this study, AGE enhanced death of RMC. Both L-165,041 (Fig. 5a) and exendin (Fig.5b) significantly attenuated AGE-induced cell death. The inhibitory action of L-165,041 was reversed by siRNA of PPARδ (Fig. 5a). No synergic effect between L-165,041 and exendin in inhibiting AGE-induced cell death was noted. L-165,041 or exendin alone showed no influence on cell survival in the absence of AGE.

## Discussion

This study is the first investigation to show anti-inflammatory effect of GLP-1 receptor agonists on AGE-stimulated rat mesangial cells and to compare the dose potency between GLP-1 receptor and PPARδ agonists. GLP-1 had been shown to inhibit AGE-induced inflammation in various cell lines. With a concentration of 0.3 nM, GLP-1 inhibited nonglycated bovine serum albumin (BSA)-induced RAGE expression, AGE-induced oxidative stress and cytokine expression in human mesangial cells [10], kidney proximal tubular epithelial cells [13], and umbilical vein endothelial cells [14]. In pancreatic beta cell line HIT-T 15, 10 nM GLP-1 improved AGE-induced cell death, oxidative stress, impaired insulin secretion, and RAGE expression [15]. In our study, the inhibitory of RAGE expressions by exendin-4 and L-165,041 incubation were reversed by siRNA of PPARδ treatment. We suggest that AGE may not only induce the RAGE expressions but also PPARδ expressions in RMC.

Few papers showed anti-inflammatory effect of GLP-1 receptor agonists on cultured cells. Exendin-4 with a concentration of 0.3 nM was shown to inhibit high glucose-induced transforming growth factor-β1 (TGF-β1) and CTGF in human mesangial cells [11]. In this study, 0.3 nM exendin-4 showed similar anti-inflammatory potency on RMC to 1 μM L-165,041. In previous studies, 1 μM L-165,041 was able to decrease CRP-induced IL-6 expression in rat cardiomyocytes [16] and human umbilical vein endothelial cells [17], and shared similar potency to another PPARδ agonist, troglitazone (10 μM), in inhibiting high glucose (25 mM)-induced inflammation and apoptosis in mesangial cells [18]. GLP-1 receptor agonists and dipeptidyl peptidase IV (DPP-4) inhibitors are being widely used in clinical treatment. These findings in dose potency are valuable for future use and development of antidiabetic agents.

The molecular mechanisms responsible for anti-inflammatory effect of GLP-1 are still under investigation. Various mechanisms have been proposed [19]. Previous studies showed that GLP-1 inhibited apoptosis of insulin-secreting cells via cyclic 5′-adenosine monophosphate (cAMP) [20]. Cyclic AMP activates protein kinase A (PKA) and increases the expression of numerous genes via PKA-mediated phosphorylation of a cAMP-responsive transcriptional factor, CREB (cAMP response element-binding protein) [21]. The cAMP-PKA-CREB axis and activated substances may be the major signal pathway responsible for anti-inflammatory effect of GLP-1. Hyperglycemia increases NADPH oxidase, and upregulates renal superoxide production. NADPH oxidase-induced superoxide production was shown to be reduced by cAMP and subsequent PKA [22]. Oxidative stress in vascular smooth muscle cells from spontaneously hypertensive rats was enhanced by reducing cAMP levels [23]. In this study, we demonstrated that PPARδ agonist and GLP-1 agonist significantly decreased Nox4 expressions and increased the PKA activity. A DPP-4 inhibitor, sitagliptin, was recently shown to improve pancreatic islet vascularization through activating CREB and subsequent VEGF-A/VEGFR-2 signal pathway [24]. An analogue of cAMP, 8-Br-cAMP, induced similar effect as GLP-1 in human mesangial cells [10].

HO-1 is one of the substances activated through cAMP-PKA-CREB signal pathway [25]. GLP-1 and GLP-1 receptor agonists increased HO-1 expression and decreased oxidative stress in rat endothelial [26] and pheochromocytoma (PC12) cells [27], respectively. In a rat model of renal ischemia-reperfusion injury, exendin-4 significantly increased renal HO-1 expression, decreased kidney injury, caspase-3 expression, and apoptosis [28]. Our results indicated that both L-165,041 and/or exendin treatment significantly increased HO-1 expression after AGE treatment. SREBP-1c/Caveolin-1 signaling was proved to involve in PPARδ-regulated GLP-1R expression and GLP-1R-dependent Akt/bcl-2 signaling were related to PPARδ agonist treatment in pancreatic beta cell [29]. We suggest that PPARδ agonist downstream signaling may correlated to GLP-1R signaling in kidney cell. Further studies are needed to identify the downstream substances of cAMP-PKA-CREB axis following GLP-1 administration and the synergic effects with PPARδ agonists.

A number of case reports showed acute kidney injury in patients receiving exenatide, a modified peptide of GLP-1, injection for glucose control [30]. Some of these patients suffered from irreversible kidney function impairment after stopping exenatide treatment [31]. In our study, exendin-4 prevents AGE-induced cell death and no cell injury was noted. Kidney injury in clinical observation may be due to indirect mechanisms of exenatide. However, the downstream signaling and effects of GLP-1 peptide treatment in human mesangial cells should be more studied. The safety and renoprotective effect of GLP-1 receptor agonists need to be identified by large-scale clinical studies.

## Conclusions

In conclusion, PPARδ and GLP-1 receptor agonists inhibit AGE-induced RAGE expressions and mesangial cell death. The renoprotective effects of PPARδ and GLP-1 receptor agonist treatments may due to the anti-inflammatory and anti-oxidative effects. The siRNA of PPARδ reversed the exendin-decreased RAGE expressions. It is suggested that GLP-1R downstream signaling may correlate to PPARδ activation. Further studies are necessary to identify the actual mechanism of GLP-1 administration and the synergic effects with PPARδ agonists treatment.

## Abbreviations

AGEs: Advanced glycation end products
Exendin-4: GLP-1 receptor agonist
GLP-1: Glucagon-like peptide-1
L-165,041: PPARδ agonists
PPARδ: Peroxisome proliferator-activated receptor-delta
RAGE: Advanced glycation end products receptor for AGEs
RMC: Rat mesangial cells
