# Correction: Glucagon-like peptide receptor agonists attenuate advanced glycation end products-induced inflammation in rat mesangial cells

**DOI:** 10.1186/s40360-023-00688-5

**Published:** 2023-10-27

**Authors:** Jui-Ting Chang, Yao-Jen Liang, Chia-Yu Hsu, Chao-Yi Chen, Po-Jung Chen, Yi-Feng Yang, Yen-Lin Chen, Dee Pei, Jin-Biou Chang, Jyh-Gang Leu

**Affiliations:** 1 Division of Nephrology, Department of Internal Medicine, Shin Kong Wu Ho-Su Memorial Hospital, Taipei, Taiwan; 2 Department and Institute of Life Science, Fu-Jen Catholic University, New Taipei, Taiwan; 3 Graduate Institute of Institute of Applied Science and Engineering, Fu-Jen Catholic University, New Taipei, Taiwan; 4 Department of Pathology, Cardinal Tien Hospital, Medical School, Fu Jen Catholic University, New Taipei City, Taiwan; 5 Division of Endocrinology and Metabolism, Department of Internal Medicine, Cardinal Tien Hospital, Medical School, Fu Jen Catholic University, New Taipei City, Taiwan; 6 Fu-Jen Catholic University School of Medicine, No. 510, Zhongzheng Road, Xinzhuang District, 24205 New Taipei City, Taiwan; 7 Department of Pathology, Division of Clinical Pathology, National Defense Medical Center, Tri-Service General Hospital, Taipei, Taiwan; 8 Department & Institute of Pharmacology, National Yang Ming University, Taipei, Taiwan

**Correction to: BMC Pharmacol Toxicol 18, 67 (2017).**

10.1186/s40360-017-0172-3

In the original publication of this article the following affiliation was missing for Jui-Ting Chang:

- Department & Institute of Pharmacology, National Yang Ming University, Taipei, Taiwan

The article has been updated to add the missing affiliation.

